# Heme Oxygenase-1: A Critical Link between Iron Metabolism, Erythropoiesis, and Development

**DOI:** 10.1155/2011/473709

**Published:** 2011-11-20

**Authors:** Stuart T. Fraser, Robyn G. Midwinter, Birgit S. Berger, Roland Stocker

**Affiliations:** ^1^Laboratory for Blood Cell Development, Disciplines of Physiology, Anatomy and Histology, School of Medical Sciences and Bosch Institute, University of Sydney, Medical Foundation Building, 92-94 Parramatta Road, Camperdown, NSW 2050, Australia; ^2^Center for Vascular Research, Discipline of Pathology, School of Medical Sciences and Bosch Institute, University of Sydney, Medical Foundation Building, 92–94 Parramatta Road, Camperdown, NSW 2050, Australia

## Abstract

The first mature cells to arise in the developing mammalian embryo belong to the erythroid lineage. This highlights the immediacy of the need for red blood cells during embryogenesis and for survival. Linked with this pressure is the necessity of the embryo to obtain and transport iron, synthesize hemoglobin, and then dispose of the potentially toxic heme via the stress-induced protein heme oxygenase-1 (HO-1, encoded by *Hmox1* in the mouse). Null mutation of *Hmox1* results in significant embryonic mortality as well as anemia and defective iron recycling. Here, we discuss the interrelated nature of this critical enzyme with iron trafficking, erythroid cell function, and embryonic survival.

## 1. Iron, Heme, and Hemoglobin

Adult humans require 4-5 mg of iron per day [1]. The predominant use of iron is in its reduced ferrous state (Fe^2+^) in heme complexed with two *α*-globin and two *β*-globin chains to form hemoglobin. Reduced iron in heme is uniquely amenable for the transport of oxygen and carbon monoxide, while hemoglobin can also bind allosteric ligands such as carbon dioxide and nitric oxide. When red blood cells are lysed, iron in the released hemoglobin can become oxidized to its ferric (Fe^3+^) form, and heme no longer binds tightly to hemoglobin and may be released [2, 3]. The hydrophobicity of the resulting free heme allows it to cross cell membranes leading to oxidative stress within cells [4, 5]. If left uncontrolled, heme and its iron can contribute to the cellular labile iron pool and act as a pro-oxidant by catalytically amplifying the production of oxidants inside the cell via Fenton chemistry whereby Fe^2+^ is oxidized by hydrogen peroxide to Fe^3+^, a hydroxyl radical, and hydroxyl anion. Amplification of oxidant production by redox-active iron can directly lead to lipid, protein, and DNA damage and ultimately cell death. However, free iron can be rapidly neutralized by a number of metabolic pathways induced by the iron itself. These include the induction of iron efflux systems or upregulation of ferritin, a cytosolic protein that sequesters iron in a redox-inactive form, thereby limiting its pro-oxidant capacity. Free heme has also been shown to have proinflammatory properties. With the vast amount of heme used in erythroid cells, mechanisms must be put into place to allow the body to cope with free iron and heme released during red blood cell clearance in the spleen.

## 2. Heme Catabolism

**Figure 1:**
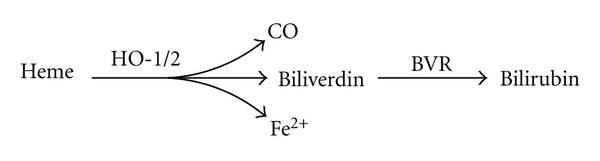
Heme catabolism initiated by heme oxygenases. Heme oxygenase-1 and 2 (HO-1/2) degrade heme to carbon monoxide (CO), Fe^2+^, and biliverdin that is then reduced to bilirubin by biliverdin reductase (BVR).

Heme oxygenases are the initial and rate-limiting enzymes in the breakdown of heme (iron protoporphyrin IX) that itself plays an essential role in the transport of oxygen and mitochondrial electron transport as a cofactor of hemoglobin, myoglobin, and cytochromes. Degradation of heme generates carbon monoxide, iron, and biliverdin, the latter of which is subsequently converted to bilirubin by biliverdin reductase (Figure 1). Heme oxygenases bind heme in a 1 : 1 molar complex where turnover of each heme molecule requires three molecules of O_2_ and seven electrons derived from NADPH supplied by cytochrome P450 reductase. Heme oxygenases are unusual in their ability to use heme both as a substrate and prosthetic group for its own degradation. However, heme oxygenases are not heme proteins in the classic sense of the cytochromes or peroxidases [6]. The removal of toxic heme is not the only function of heme oxygenases, as the products of their enzymatic activity have now being recognized to play significant roles in vascular biology, iron recycling, and cellular protection against oxidative stress and diseases associated with such stress [7, 8].

Heme oxygenases are evolutionarily conserved, and various forms can be detected in bacteria, plants, fungi, and animals indicating that the need for heme degradation occurred early during evolution. A heme oxygenase “fingerprint” motif has been used to identify heme-degrading enzymes from a range of organisms. Bacterial and human heme oxygenases show up to 70% homology within this motif [9]. There are two distinct isoforms of heme oxygenase, the inducible heme oxygenase-1 (HO-1) and a constitutively expressed form, HO-2 [10]. HO-1 and HO-2 are the products of different genes, and the amino acid sequences of the human HO-1 and HO-2 isoforms show ~42% sequence homology [11]. While both isoforms require similar substrates and cofactors for heme oxidation, the kinetics of the enzymatic reaction differs with the *K*_*m*_ value for heme being three times higher for HO-2 than HO-1. 

HO-1 is a 32-kDa protein also known as heat shock and stress response protein HSP32. HO-1 is an early stress response gene and the predominant heme oxygenase species present in the spleen under physiological conditions [11]. High levels of HO-1 are detected in tissues that degrade aged red blood cells such as the specialized reticuloendothelial cells of the spleen, liver, and bone marrow [7]. In most tissues not directly involved in erythrocyte or hemoglobin turnover, HO-1 is generally not detected. However, a diverse range of chemical and physical stresses and treatments, such as heme, cytokines, lipopolysaccharide, growth factors, heat shock, hydrogen peroxide, and heavy metals, rapidly induce HO-1. This induction is believed to indicate a critical adaptive response to cellular stress.

HO-1 localizes to the ER, anchored there by a single hydrophobic transmembrane spanning region at the C-terminal domain. HO-1 localizes in close proximity to NADPH cytochrome P450 reductase which is required for maximal enzymatic activity [12]. However, recently HO-1 has been demonstrated to localize to other subcellular organelles including the plasma membrane, mitochondria, and the nucleus [13, 14]. While the biological implications of this sub-cellular compartmentalization have not been fully elucidated, it raises the possibility of organelle-specific function of HO-1 and its metabolites [12]. 

## 3. Erythropoiesis throughout Life

**Figure 2:**
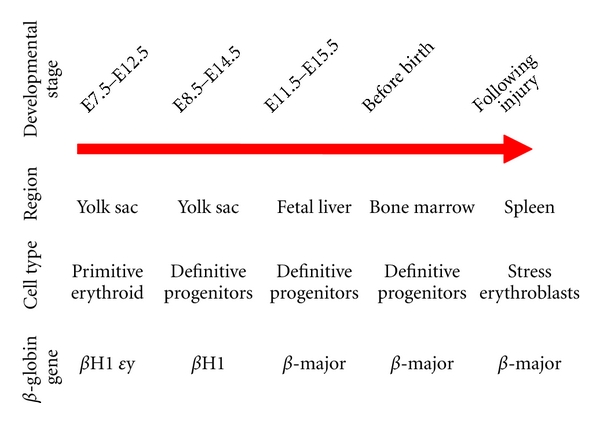
Distinct waves of erythroid cell generation in the mouse. Different populations of erythroid cells are generated during distinct embryonic periods in the bone marrow and following injury in the mouse. These populations vary according to their site of origin, embryonic stage of production, morphology, and *β*-globin gene expression. E: embryonic day, scored from days following coitus.

The erythroid or red blood cell lineage is the main consumer of iron and heme in the human body. This lineage is also the first cell type to mature during mammalian embryogenesis [15]. In mice, at least five distinct forms of erythropoiesis, or red blood cell production, have been identified (Figure 2). The erythrocytes produced during these distinct waves may vary according to cell size and globin gene expression profile, but all serve to transport oxygen and metabolic waste products via hemoglobin. Systems regulating both heme production and catabolism are, therefore, required from early embryonic development and throughout adulthood to prevent the release of free heme and subsequent oxidative stress.

 The first erythroid population to arise is the primitive erythroid lineage [16]. These red blood cells are up to 6-times larger than their adult counterparts and express a unique set of globin genes only activated during embryogenesis [15, 17]. These cells are generated in the extraembryonic yolk sac, dominate the embryonic circulation, but are almost undetectable by birth [17, 18]. The yolk sac is also the source of another, recently identified erythroid population which express a mixture of embryonic and adult-type globin genes and are thought to populate the fetal liver [19]. The fetal liver itself gives rise to a third distinct population which expresses only adult-type globin genes. Shortly before birth, the bone marrow becomes the predominant site of life-long blood cell production. These erythrocytes express adult globin genes only and are slightly smaller than those produced by the fetal liver. An auxiliary wave of erythropoiesis is generated following stress, such as significant blood loss or ongoing erythroid disease such as sickle cell anemia or *β*-thalassemia. This wave is termed stress erythropoiesis and is characterized by migration of immature erythroblasts from the bone marrow to the spleen [20]. Regulation of stress erythropoiesis is a research area that has only recently obtained some of the interest it warrants, and the processes regulating this phenomenon are ill defined.

## 4. Macrophage Function during the Erythroid Cell Lifecycle

Macrophages play crucial roles in erythropoiesis from the cradle of the bone marrow to the grave of the spleen. In erythropoietic tissues, such as the fetal liver and bone marrow, erythroblasts are found in intimate proximity to supportive macrophages in multicellular structures termed erythroblastic islands (EBIs) [21]. These structures are thought to play crucial roles in regulating erythroid cell production with a central macrophage often interacting with numerous erythroblasts [22]. Central EBI macrophages have been postulated to transfer iron to the developing erythroblasts to complete hemoglobin synthesis. This idea is strengthened by the initial description of EBI macrophages as containing iron detected by Perls' staining [21]. However, developing erythroblasts also express high levels of the transferrin receptor, CD71, demonstrating the vast appetite for iron that these cells have [23, 24].

Central EBI macrophages are critical for the engulfment and destruction of the vast numbers of erythroid cell nuclei that are expelled in the final stages of red blood cell maturation. Evidence for the uptake of expelled erythroid nuclei comes from several sources. Fluorescently tagged, primitive erythroid nuclei have been observed engulfed by fetal liver macrophages [25]. Secondly, null mutation of DNAse II, the major DNA-degrading enzyme present in macrophages, results in the massive buildup of erythroid nuclei within the cytoplasmic of fetal liver macrophages [26]. As DNAse II-deficient macrophages are unable to degrade erythroid DNA, these phagocytes swell from engulfed nuclei, rupture, and release inflammatory mediators into the fetal liver killing the embryo *in utero *[26].

Macrophages also play a critical role at the end of the erythrocyte lifespan. Aged or damaged erythrocytes are removed from the circulation by macrophages found in the red pulp of the spleen. Macrophages recognise aged erythrocytes by increased expression of surface phosphatidylserine and by decreased levels of the “self” antigen CD47 [27, 28]. Indeed, one of the chief roles of splenic macrophages is to engulf and destroy aged or damaged erythrocytes, catabolize the heme from the hemoglobin, and return the free iron to the developing erythroblasts of the bone marrow via transferrin [1].

## 5. The Impact of HO-1 Deficiency in Mice and Humans

Heme oxygenase-1-deficient mice have been generated by targeted null mutation of the *Hmox1* gene. The original authors, other groups, and our own group (unpublished data) have noticed abnormally low number of *Hmox1^−/−^* mice at birth, strongly suggesting that the *Hmox1 *deficiency is lethal in a significant number of embryos [29, 30]. Approximately 20% of the expected number of *Hmox1^−/−^* pups are born live [29]. The infrequent *Hmox1^−/−^* animals that survive until adulthood are smaller in size compared with their littermate wild-type mice and exhibit a range of pathological conditions including splenic, hepatic, and renal fibrosis as well as increased expression of indicators of stress and inflammation [31]. Accumulation of iron is observed in tissues throughout the body of *Hmox1^−/−^* mice, strongly suggesting that these animals suffer from defects in the trafficking of iron from peripheral tissues back to sites of erythropoiesis [32].

Heme oxygenase-1 deficiency in humans appears to be an extremely rare condition with only two live births reported [33, 34]. Both patients presented with severe anemia [33, 34]. The patient reported in Japan showed a high degree of erythrocyte fragmentation as well as high serum concentrations of heme, iron deposition, and a striking absence of a spleen [33]. This latter condition was distinct from the phenotype seen in adult *Hmox1^−/−^* mice, which exhibit enlarged spleens [29]. As heme is a potent inducer of oxidative stress, it has been postulated that most of the pathophysiological conditions observed in these extremely rare individuals, including hyperlipidemia, vascular disease, and renal injury, are due to the inability to rid the body of heme [35].

## 6. Heme Oxygenase-1 and Embryonic Survival

Embryonic survival appears to be linked to HO-1 gene dosage. The mother of the Japanese HO-1-deficient patient was found to be heterozygous for *Hmox1* and had experienced several fetal deaths [33]. Supporting this, polymorphisms in the human *HMOX1 *gene have been associated with an increased risk for idiopathic recurrent miscarriage [36]. HO-1 is expressed widely throughout the placenta including the syncytiotrophoblasts and endothelial cells [37]. Carbon monoxide produced locally by heme catabolism by HO-1 is thought to contribute to the regulation of vascular tone within the placenta. HO-1 expression is also thought to stimulate trophoblast invasion into the placenta [38]. To investigate possible placentation defects, Zhao and colleagues examined *Hmox1^+/−^* embryos and found that their placentae were reduced in weight [30]. This was attributed to apoptosis in the spongiotrophoblast layer. HO-1 expression during ontogeny has been examined in the rat, where the enzyme is expressed in the syncytiotrophoblasts of the placenta as well as the endodermal layer of the yolk sac [39]. Expression was also detected in immature macrophages in the yolk sac and later in macrophages in the fetal liver [39]. Similarly, during the first 48 hours of development of the zebra fish embryo, Hmox1 mRNA was detected in yolk syncytial layer, blood cells, as well as the lens [40]. *Hmox1 *deficiency may, therefore, affect development of the mouse yolk sac through changes in the endoderm, as well as resulting in macrophage defects. Collectively, these data implicate roles for HO-1 in placenta formation, development of the yolk sac, and function of macrophages in the embryo itself. Interference with any of these developmental steps could lead to embryonic lethality.

## 7. Heme Oxygenase-1 and Hematopoiesis

The spleen encounters the greatest amount of heme, arising from the clearance of aged or damaged erythrocytes by red pulp macrophages. Spleens from *Hmox1^−/−^* mice are enlarged and show decreased amounts of free iron, as assessed by Perls Prussian blue staining [32]. Macrophages isolated from such spleens are reduced in number and show intoxication from heme. These macrophages are able to engulf and destroy aged or damaged red blood cells. However, they fail to detoxify the heme released from the breakdown of the engulfed erythrocytes due to the lack of *Hmox1 *[32]. This is thought to lead to macrophage death and splenomegaly. Spleens from *Hmox1^−/−^* mice are also highly fibrotic, again a defect most likely associated with the lack of macrophages. This results in profound changes in iron recycling. In the wild-type setting, macrophages in the spleen and liver are the main iron trafficking cells. In the absence of HO-1, proteins that serve to sequester free hemoglobin and heme (i.e., haptoglobin and hemopexin, resp.) are elevated in the liver and kidney [32]. In the case of haptoglobin, this may be a consequence of the drastic decrease in the expression of CD163, the scavenger receptor that normally removes haptoglobin-hemoglobin complexes from the circulation [32]. Instead, hepatocytes and the proximal tubular epithelial cells of the kidney become the predominant cell types regulating the reutilization of iron derived from erythrocytes in HO-1*^−/−^* mice, likely relying on the enzymatic activity of HO-2 [32]. This suggests that compensatory pathways are enhanced in the absence of the main system to detoxify the body of heme.

*Hmox1* gene dosage has been implicated in the response of the hematopoietic system to stress. *Hmox1^+/−^* mice treated with the hematopoietic toxin 5-fluorouracil showed a more rapid recovery in blood cell counts compared with wild-type mice [41]. However, this response to stress was not observed during transplantation assays where hematopoietic stem cells from *Hmox1^+/−^* mice poorly reconstituted irradiated adult host animals and failed to give rise to serially transplantable hematopoietic stem cells [41]. The same group also found that irradiated adult mice, reconstituted with *Hmox1^+/−^* bone marrow cells, showed significant defects in the trafficking of erythroblasts to the spleen. While this is a relatively artificial stress model, these results do suggest that erythroblasts migrating from the bone marrow to the spleen may be influenced by *Hmox1* gene dosage [42].

## 8. Unresolved Issues

One of the most intriguing questions in this field is why most *Hmox1^−/−^* embryos die *in utero* while a small fraction are born live and can survive until late adulthood? Knockout mice lacking genes essential for erythropoiesis are often 100% embryonic lethal (e.g., all *Eklf*-deficient embryos die *in utero *[43]) or survive until adulthood with milder defects. A phenotype resulting in most embryos dying *in utero* with a small percentage surviving until late adulthood is, therefore, highly unusual. The fact that a small number of *Hmox1^−/−^* mice and HO-1-deficient individuals are born live suggests that mechanisms, such as genetic or environmental modifiers, can rescue these defects sufficiently to allow survival until birth. Is the lethality seen in HO-1 knockout embryo due to death of macrophages intoxicated by heme they are unable to catabolize following engulfment of embryonic erythroid cells? Or is HO-1 playing a more important role in epithelial cells such as the syncytiotrophoblasts or visceral endoderm of the yolk sac? Alternatively, environmental factors derived from the mother may somehow rescue this typically lethal phenotype. Resolving these modifiers will shed light onto the function of HO-1. A clear reason for the survival of a fraction of the HO-1*^−/−^* mice to birth and even one year of age, while others die *in utero*, is still not readily apparent. 

## 9. Concluding Remarks

During evolution, the utility of both iron and heme has been harnessed to drive basic biochemical activities within our cells. Erythroid cells, in particular, have used these compounds extremely effectively to transport gases throughout our bodies. However, this is a double-edged sword as the very activities that make iron and heme useful are the same that make them potentially toxic to our cells. HO-1 is essential for ameliorating its toxicity. This is highlighted by the embryonic lethality of HO-1 deficiency. The activity of this enzyme is, therefore, a critical tipping point between health and anemia or embryonic death. Increasing our knowledge of the roles of HO-1 in embryonic development may provide a better understanding of how the enzyme provides protection against oxidative stress-associated diseases.
